# Benefits of non-medical prescribing within an NHS podiatry service

**DOI:** 10.1186/1757-1146-3-S1-O20

**Published:** 2010-12-20

**Authors:** Susan Popadiuk, Satyan Rajbhandari

**Affiliations:** 1 NHS Central Lancashire, Preston, UK

## Introduction

May 2005 saw the publication of the supplementary framework within which podiatrists can prescribe. The incorporation of supplementary prescribing into existing podiatry pathways was initiated within the podiatry Service

## Method

A comprehensive history was taken initially and then a draft Clinical Management Plan (CMP) was produced during the patient’s podiatry appointment. The CMP was then discussed and agreed at the patient’s multidisciplinary foot clinic appointment. The patient’s medication, compliance and supply prescriptions are checked during their follow- up uniprofessional podiatry appointments. All prescriptions given, CMPs agreed and the patient’s main medical history are inputted onto a spread sheet for auditing.

## Results

**Figure 1:**
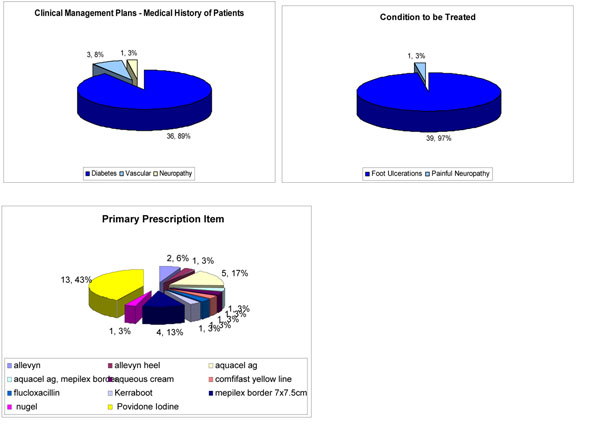


Uncorroborated reduction in the frequency of clinic visits and secondary care visits has been found. However, from the audit undertaken the following was found - 89% of the CMPs were for patients with diabetes, 8% patients with peripheral vascular disease and 3% for patients with neuropathy. Prescriptions fell into 11 main groups with 97% for dressings and 3% for antibiotics (Figure 1).

## Conclusion

Patients only require an initial assessment by the Independent Practitioner, for which they need to attend secondary care. Podiatrists can prescribe according to their CMP in primary care ensuring seamless provision of care across primary and secondary care. Patients are encouraged to bring their dressings to the clinic to ensure continuity and avoid confusion regarding dressings used. Patients who have their own dressings are empowered to take control of their disease.

This system has been used to improve Painful Diabetic Neuropathy patient's access to treatment and monitor their medication in primary care.

This evidence has been accepted onto the NHS evidence web site, through SCP.
